# Protective Effect of Piceatannol Against Acute Lung Injury Through Protecting the Integrity of Air-Blood Barrier and Modulating the TLR4/NF-κB Signaling Pathway Activation

**DOI:** 10.3389/fphar.2019.01613

**Published:** 2020-01-22

**Authors:** Lu-Yuan Peng, Meng Yuan, Hai-Tao Shi, Jing-He Li, Ke Song, Jiang-Ni Huang, Peng-Fei Yi, Ben-Dong Fu, Hai-Qing Shen

**Affiliations:** College of Veterinary Medicine, Jilin University, Changchun, China

**Keywords:** acute lung injury, lipopolysaccharide, piceatannol, air-blood barrier, TLR4/NF-κB

## Abstract

Acute lung injury (ALI) is a common and complex inflammatory lung syndrome with higher morbidity and mortality rate. Piceatannol (PIC) has anti-inflammation and anti-oxidant properties. The study was designed to explore the effect and the action mechanisms of PIC on lipopolysaccharide (LPS)-induced ALI. Twenty-four hours after LPS challenge, mice from different treatment groups were euthanized, and the bronchoalveolar lavage fluid (BALF) and lung tissue samples were collected. Then the degree of pulmonary edema, lung pathological changes, myeloperoxidase (MPO) activity, and the production of pro-inflammatory cytokines were detected. Additionally, the messenger RNA (mRNA) expressions associated with cell adhesion molecules and tight junction were analyzed through quantitative real-time (qRT)-PCR, and the TLR4/NF-κB activation was examined by western blot. The results showed that PIC significantly inhibited LPS-induced lung edema, histopathological damage, MPO activity, cell infiltration, and pro-inflammatory cytokines production. Moreover, PIC notably suppressed mRNA expressions associated with inflammation and cell adhesion molecules. Furthermore, PIC also alleviated LPS-induced damage of air-blood barrier through reducing the levels of total proteins in BALF and recovering the expression of occludin and ZO-1 in the lung tissues. We also found that PIC remarkably restrained the LPS-induced TLR4/NF-κB pathway activation in lung tissues. In conclusion, PIC may be potential to treat LPS-induced acute lung injury (ALI) *via* regulating air-blood barrier and TLR4/NF-κB signaling pathway activation.

## Introduction

Acute lung injury (ALI), known as pulmonary inflammation, is a life-threatening clinical condition due to its higher morbidity and mortality worldwide (Ji et al., 2019; Khan et al., 2019). The ALI mainly characterizes with cell infiltration, cytokine production, air-blood barrier permeability increase, pulmonary edema, and pathological damage (Pinheiro et al., 2019). Lipopolysaccharides (LPS)-induced ALI model in mice is usually applied to mimic the pathological process of ALI. In the process of ALI, LPS induced serve inflammatory response in lung and activated the TLR4 signal pathway as well as NF-κB signal pathway to regulate the infiltration of inflammatory cells and the production of pro-inflammatory cytokines (Wu et al., 2019). In addition, LPS can break the integrity of air-blood barrier and induced accumulation of the protein-rich fluids in the bronchoalveolar lavage fluid (BALF) leading to pulmonary edema and hypoxemia (Zhang et al., 2015). Although many studies have focused on the treatment and therapeutic strategies for ALI, the mortality of ALI is still approximately 40% (Frutos-Vivar et al., 2006). Therefore, exploring effective anti-inflammation agents for the treatment of ALI are urgently needed.

Piceatannol (PIC), a structurally related analog of resveratrol, is widely isolated from blueberries, grapes, and passion fruit seeds, etc. PIC has been proved to possess anti-inflammatory, anti-oxidative, anti-proliferative activities (Kalariya et al., 2013). Moreover, it has been used to treat many diseases including liver injury (Wen et al., 2018), cardiac injury (Wang et al., 2019), cancer (Lucas et al., 2018) and skin disease (Maruki-Uchida et al., 2018). Evidence indicated that treatment of PIC visibly repressed the secretion of pro-inflammatory cytokines and nitric oxide (NO) in RAW264.7 cells during LPS stimulation (Yamamoto et al., 2017). Further study showed that PIC suppressed the production of NO due to its inhibition of P13k/Akt phosphorylation and IKK/IκB activation (Chiou et al., 2011). In addition, other studies also showed that treatment with PIC down-regulated the production of pro-inflammatory cytokine through regulating NF-κB signaling pathway activated by LPS both in BV2 cells and human myeloid cells (Ashikawa et al., 2002; Jin et al., 2006). However, the potential effect of PIC on the ALI has not been reported. Thus, we aimed to investigate the protective effects of PIC against LPS-induced ALI in mice in the present study.

## Materials and Methods

### Materials

Piceatannol (purity > 95%) was purchased from Chengdu Must Biotechnology Co., Ltd. (Chengdu, China). LPS was obtained from Sigma (St. Louis, MO, USA). The indicated primary antibodies and secondary antibodies were gained from Biosynthesis Biotechnology Inc. (Beijing, China).

### Animals and Experimental Establishment

Male C57BL/6 mice (40–50 g) were supplied by Changsheng Biotechnology Co. Ltd (Liaoning, China). All animals for this experiment were kept in a temperature and humidity-controlled room with an artificial light/dark cycle and allowed to adapt the new environment for 1 week. Briefly, total 50 mice were randomly divided into five groups (*n = 10*), including the control, LPS, PIC (10, 20, and 40 mg/kg) + LPS groups. To establish ALI model, LPS (5 mg/kg) was intraperitoneal injection in mice as previous described (Yu et al., 2019). The mice of PIC group were received intraperitoneally with 10, 20, and 40 mg/kg 1 h before LPS challenge while control group mice were injected by the equal volume of saline. Twenty-four hours later, mice were euthanized and the biological samples were collected for subsequent determination. All experimental protocols were approved by the Institutional Animal Care and Use Committee of Jilin University.

### Bronchoalveolar Lavage Fluid Collection and Analysis

The BALF was harvested according to previously described (Rhee et al., 2011; He et al., 2019). Briefly, mice were euthanized and the tracheas as wells as the lung were exposed. A catheter was intubated into the trachea and BALF was extracted with a 1 ml syringe with 1 ml iced phosphate-buffered saline (PBS) of instillation and aspiration for three times. The BALF samples were centrifuged and the supernatant was collected to analyze the concentration of total protein by BCA protein assay kit (Thermo Scientific, Rockford, IL, USA). Meanwhile, the releases of tumor necrosis factor (TNF)-α and IL-1β were evaluated using commercial ELISA kits (Lengton Bioscience Company, Shanghai, China). In addition, the cell pellet was used to count the numbers of total cells, macrophages, and neutrophils using a hemocytometer.

### Lung Wet/Dry Ratio Analysis

After mice were euthanized, the fresh lung tissues were randomly selected in each group and taken from right lobes to weigh. Then, the lung lobes were put into an oven at 80°C for 48 h. Finally, the tissues were dried to weigh for the dry weight. The wet to dry ratio was calculated to evaluate the degree of lung edema.

### Myeloperoxidase Activity Analysis

The myeloperoxidase (MPO) is an indicator of the levels of polymorphonuclear neutrophils (PMN) in the infection site. The collected lung tissues were removed and homogenized with 5% extraction buffer at the ratio of 1:9 (w/v). The activity of MPO were assessed using MPO ELISA kits (Lengton Bioscience Company, Shanghai, China) according to the manufacturer's procedures. The enzymatic concentration was detected at the OD_450_.

### Pathological Analysis

A partial lung tissue of left lobes was removed and fixed in 10% formalin solution for 24 h and cut into 4–6 μm sections. Then, the slices were stained with hematoxylin and eosin (H & E) and visualized under a light microscope. In addition, lung tissue injury was scored according to previously described (Hu et al., 2019) under identical conditions.

### Pro-Inflammatory Cytokines Analysis

The concentration of pro-inflammatory cytokines including TNF-α and IL-1β in lung tissue and BALF were analyzed by the specific ELISA kits under the guidance of manufacturer's instructions (Lengton Bioscience Company, Shanghai, China).

### Quantitative Real-Time Polymerase Chain Reaction Assay

The transcriptional levels of zonula occludens-1 (ZO-1), occludin, CD11a, L-selectin (CD62L), intercellular adhesion molecule-1 (ICAM-1), monocyte chemoattractant protein−1 (MCP-1), interleukin-10 (IL-10), inducible NO synthase (iNOS), and cyclooxygenase-2 (COX-2) genes of lung tissues were detected by quantitative real-time (qRT)-PCR. In brief, the RNA was extracted by TRIzol reagent as well as the RNA quality and concentration were examined by a Q6000 trace UV-vis spectrophotometer (Quawell Technology, America). Then, 1 μg RNA was reversed to complementary DNA (cDNA) using a cDNA Synthesis SuperMix kit (TransGen Biotech, Beijing, China). The gene expression was detected using a 7500 real-time PCR system (Applied Biosystems, Foster, CA). The related primers referenced the previous studies (Ahmad et al., 2015; Chen et al., 2018a) were listed in **Table 1** and β-actin as the internal reference.

**Table 1:** Primers for quantitative real-time (qRT)-PCR.

**Primer name**	**Sequence (5' to 3')**
*ZO-1-F*	GACCTTGATTTGCATGACGA
*ZO-1-R*	AGGACCGTGTAATGGCAGAC
*Occludin-F*	ACACTTGCTTGGGACAGAGG
*Occludin-R*	AAGGAAGCGATGAAGCAGAA
*IL-10-F*	ACCTGCTCCACTGCCTTGCT
*IL-10-R*	GGTTGCCAAGCCTTATCGGA
*CD11a-F*	AACAAACCCAACGGGACAGT
*CD11a-R*	CTGCAAGCCACTCTACCTCC
*ICAM-1-F*	GCGGAGTCCGGGCAGGTCTA
*ICAM-1-R*	GGGGGCTGGCTCTGTGAGGA
*CD62L-F*	AAGGTACCGAAGGGATCCGA
*CD62L-R*	GACATGGGTGGGAACCAACA
*MCP-1-F*	ACCACAGTCCATGCCATCAC
*MCP-1-R*	TTGAGGTGGTTGTGGAAAAG
*iNOS-F*	CTATGGCCGCTTTGATGTGC
*iNOS-R*	CAACCTTGGTGTTGAAGGCG
*COX-2-F*	CACTCATGAGCAGTCCCCTC
*COX-2-R*	ACCCTGGTCGGTTTGATGTT
*β-actin-F*	GTCAGGTCATCACTATCGGCAAT
*β-actin-R*	AGAGGTCTTTACGGATGTCAACGT

### Western Blot Analysis

To further explore the anti-inflammatory activity of PIC on ALI model, the expression of TLR4/NF-κB signaling pathway in lung tissues at the protein level were accessed. The equal proteins were fractionated by 10% sodium dodecyl sulfate polyacrylamide gel electrophoresis (SDS-PAGE) gels and transferred onto polyvinylidene fluoride or polyvinylidene difluoride (PVDF) membranes. After blocking with 5% skim milk for 3 h, these membranes were incubated with primary antibody as follows: anti-TLR4, -p65, -phosphorylated-p65, -IκB, and -phosphorylated-IκB antibodies. Then, the PVDF membranes were washed by Tris buffered saline with Tween-20 (TBST) and the anti-rabbit horseradish peroxidase (HRP)-conjugated secondary antibodies were used to combine with the primary antibodies on the membranes for 2 h. Subsequently, the immunoreactive bands were visualized by the enhanced chemiluminescence solution and imaged using a ProteinSimple imager (ProteinSimple, Santa Clara, CA, USA).

### Statistical Analysis

All data were expressed as means ± SD. Tukey-Kramer multiple comparison was adopted to compare the differences of all groups using GraphPad Prism 6.0. A value of * *p* < 0.05 was considered statistically significant.

## Results

### Piceatannol Inhibited the Pulmonary Edema Induced by Lipopolysaccharide

**Figure 1:**
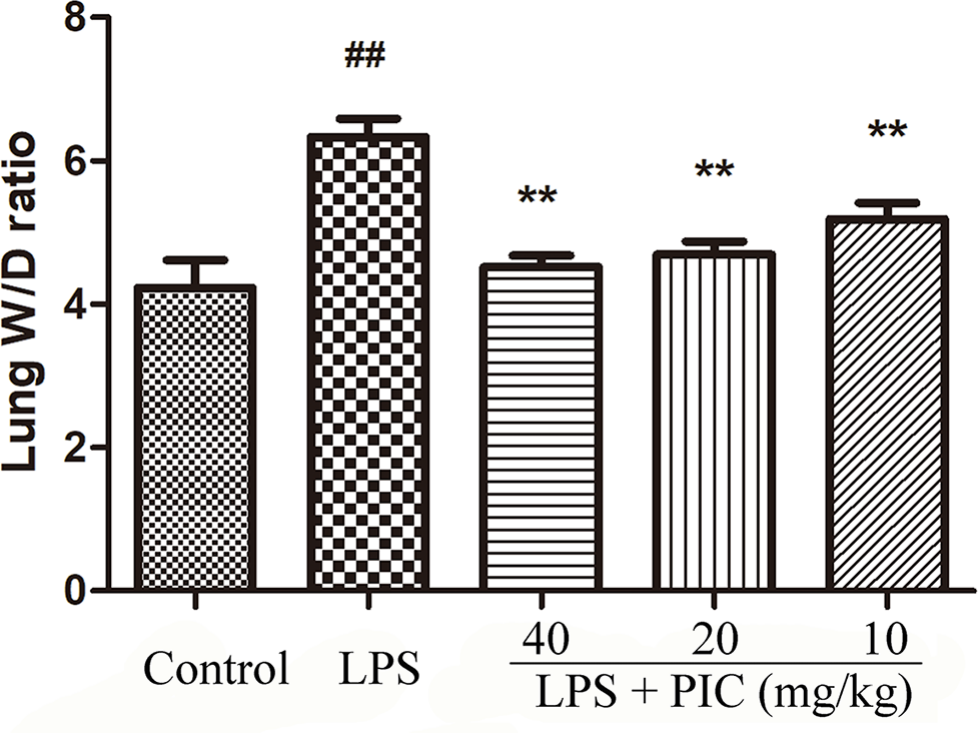
Lung wet to dry (W/D) ratio. The lung wet weight was divided by its dry weight to calculate the lung W/D ratio to evaluate the severity of pulmonary edema. ^##^ *p* < 0.01 is significantly different from the control group; **p < 0.01 are significantly different from the lipopolysaccharide (LPS) group.

The levels of alanine aminotransferase (ALT) and aspartate transaminase (AST) in serum were measured to evaluate whether PIC had toxic effect on mice. The results showed that the concentration of ALT and AST in control group and PIC treatment groups had no significant difference (**Supplementary Figure 1**). Thus, the dose of PIC at 40, 20, 10 mg/kg were safe to conduct follow-up experiments. The wet to dry ratio of lung is an important item to evaluate the pulmonary edema. In this study, we found that LPS induced prominent lung edema because of a higher wet to dry ratio compared with that of the control group mice. It was worth noting that PIC significantly reduced the pulmonary edema induced by LPS (**Figure 1**).

### Piceatannol Reduced the Infiltration of Inflammatory Cells Induced by Lipopolysaccharide

**Figure 2:**
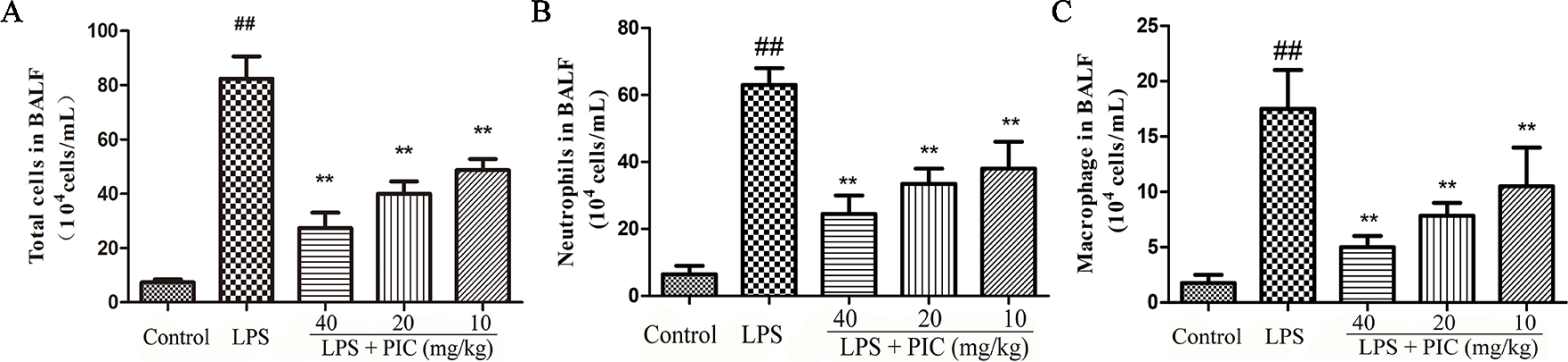
The total cells and immune cells count of bronchoalveolar lavage fluid (BALF.) After centrifuging the BALF, the cell pellet was used to count the numbers of **(A)** total cells, **(B)** neutrophil, and **(C)** macrophage by a hemocytometer. ^##^ *p* < 0.01 is significantly different from the control group; **p < 0.01 are significantly different from the lipopolysaccharide (LPS) group.

To further assess the levels of inflammatory cell infiltration, we counted the total cells, macrophage, and neutrophil in the BALF. The results showed that challenge with LPS resulted in a significant increase of total cells, macrophage, and neutrophil compared with that of the control group. In the PIC pretreatment group, the numbers of these cells were dose-dependently reduced when compared with that of the LPS treatment group (**Figure 2**).

### Piceatannol Alleviated the Myeloperoxidase Activity Induced by Lipopolysaccharide

**Figure 3:**
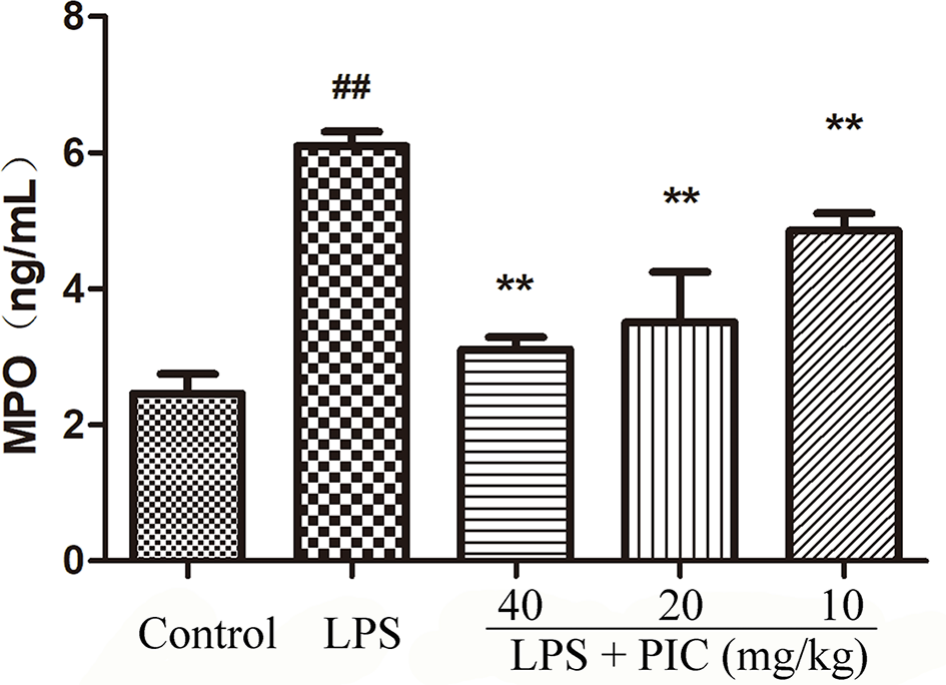
Myeloperoxidase (MPO) activity. The MPO activity in lung tissues were measured by the MPO ELISA kit. ^##^ *p* < 0.01 is significantly different from the control group; **p < 0.01 are significantly different from the lipopolysaccharide (LPS) group.

MPO activity is always regarded as a reflection of neutrophils infiltration. As shown in **Figure 3**, lung MPO activity was obviously enhanced by LPS treatment. However, PIC dose-dependently inhibited the LPS-induced MPO activity.

### Piceatannol Alleviated Lipopolysaccharide-Induced Histopathological Changes in the Lung Tissues

**Figure 4:**
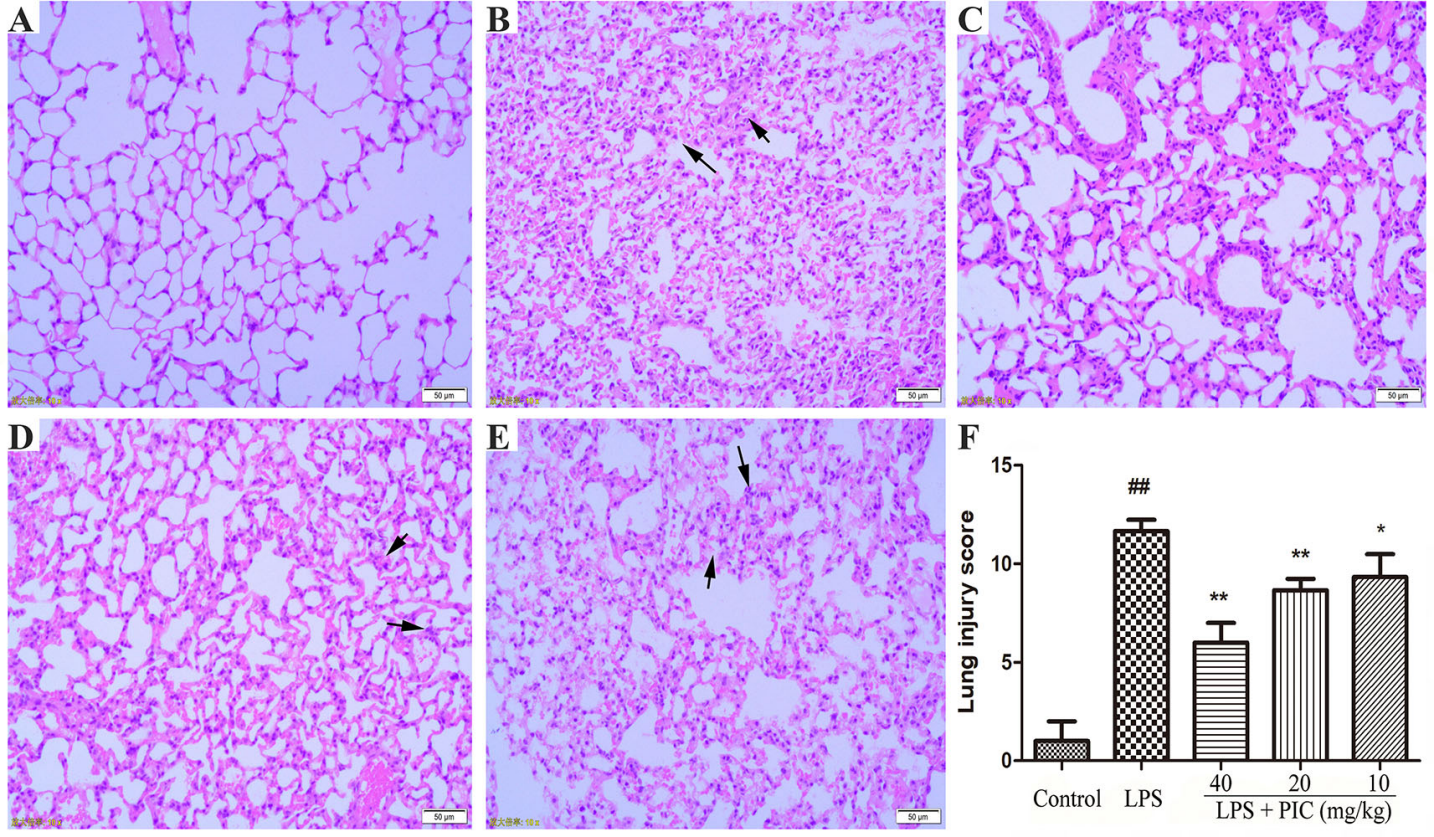
The effect of piceatannol (PIC) on lung histopathology. The lung tissues of **(A)** control group, **(B)** lipopolysaccharide (LPS) group, and **(C**–**E)** PIC (40, 20, 10 mg/kg) groups were stained with hematoxylin and eosin (H & E) and detected under a light microscope. **(F)** Then lung tissue injury was scored by a blinded assessment method for five categories. ^##^ *p* < 0.01 is significantly different from the control group; **p < 0.01 are significantly different from the lipopolysaccharide (LPS) group.

As shown in **Figure 4**, there are no abnormal changes in lung tissues from control group mice (**Figure 4A**). In the LPS treatment group mice, the lung tissues exhibited obviously pathological injury, including thickening of the alveolar wall, infiltration of inflammatory cells, and congestion (**Figure 4B**). However, treatment of PIC significantly alleviated the pathological damages of lung tissues induced by LPS (**Figures 4C–E**). Further, the LPS group mice had significantly higher lung injury score than that of control group mice. Whereas, the lung injury score of the PIC group mice was significantly lower than that of LPS group (**Figure 4F**).

### Piceatannol Suppressed the Levels of Pro-Inflammatory Cytokines in Bronchoalveolar Lavage Fluid Induced by Lipopolysaccharide

**Figure 5:**
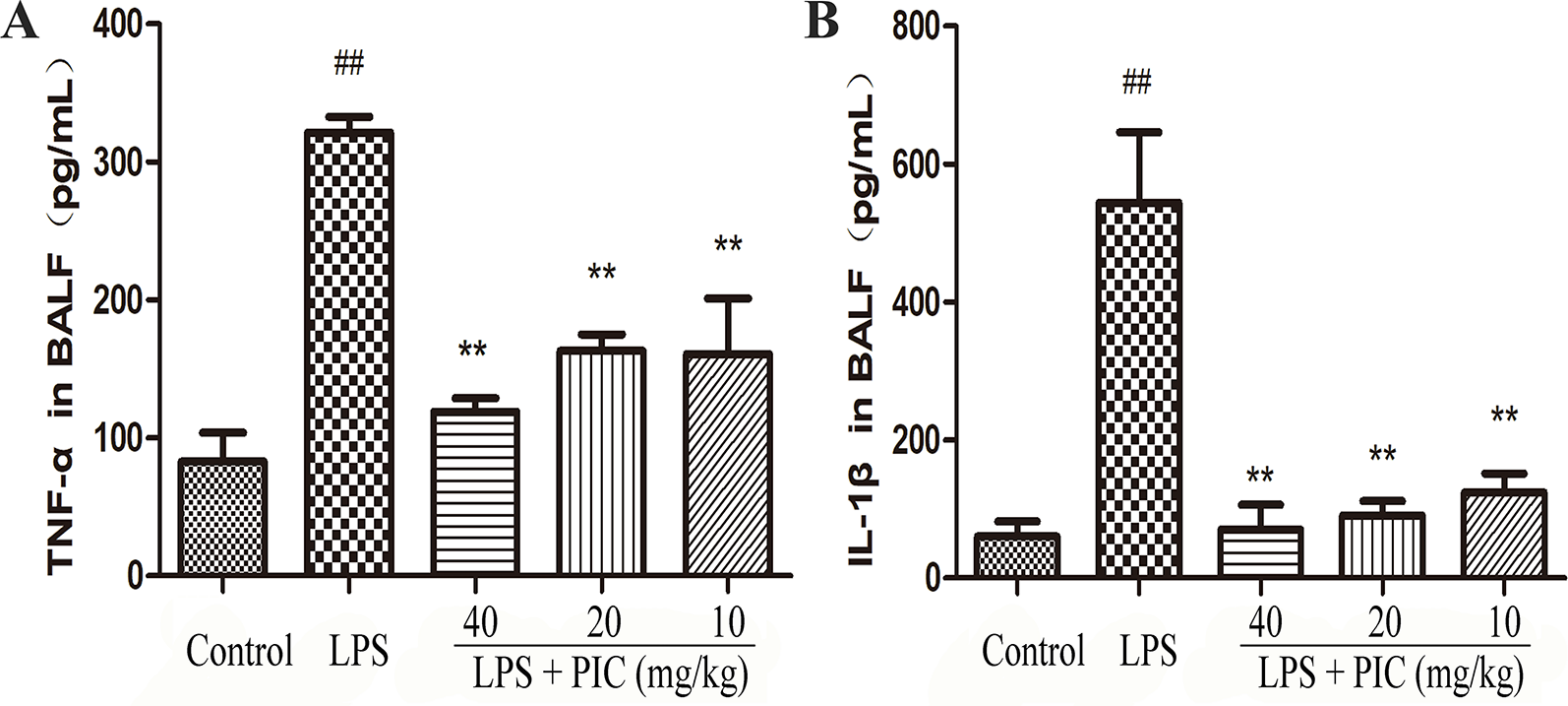
The production of pro-inflammatory cytokines in bronchoalveolar lavage fluid (BALF). After collecting the BALF, the levels of pro-inflammatory cytokines **(A)** tumor necrosis factor (TNF)-α and **(B)** interleukin (IL)-1β were detected by ELISA kits according to the manufacturer's instructions. ^##^ *p* < 0.01 is significantly different from the control group; **p < 0.01 are significantly different from the lipopolysaccharide (LPS) group.

Over-production of pro-inflammatory cytokines is important factor for the development of ALI. In this study, the data showed that challenge of LPS significantly increased the levels of TNF-α and IL-1β in the BALF when compared to those of control group mice. However, PIC dose-dependently suppressed the secretion of those pro-inflammatory cytokines in the BALF induced by LPS (**Figure 5**).

### Piceatannol Suppressed the Production of Tumor Necrosis Factor-α and Interleukin-1β in the Lung Tissues Induced by Lipopolysaccharide

**Figure 6:**
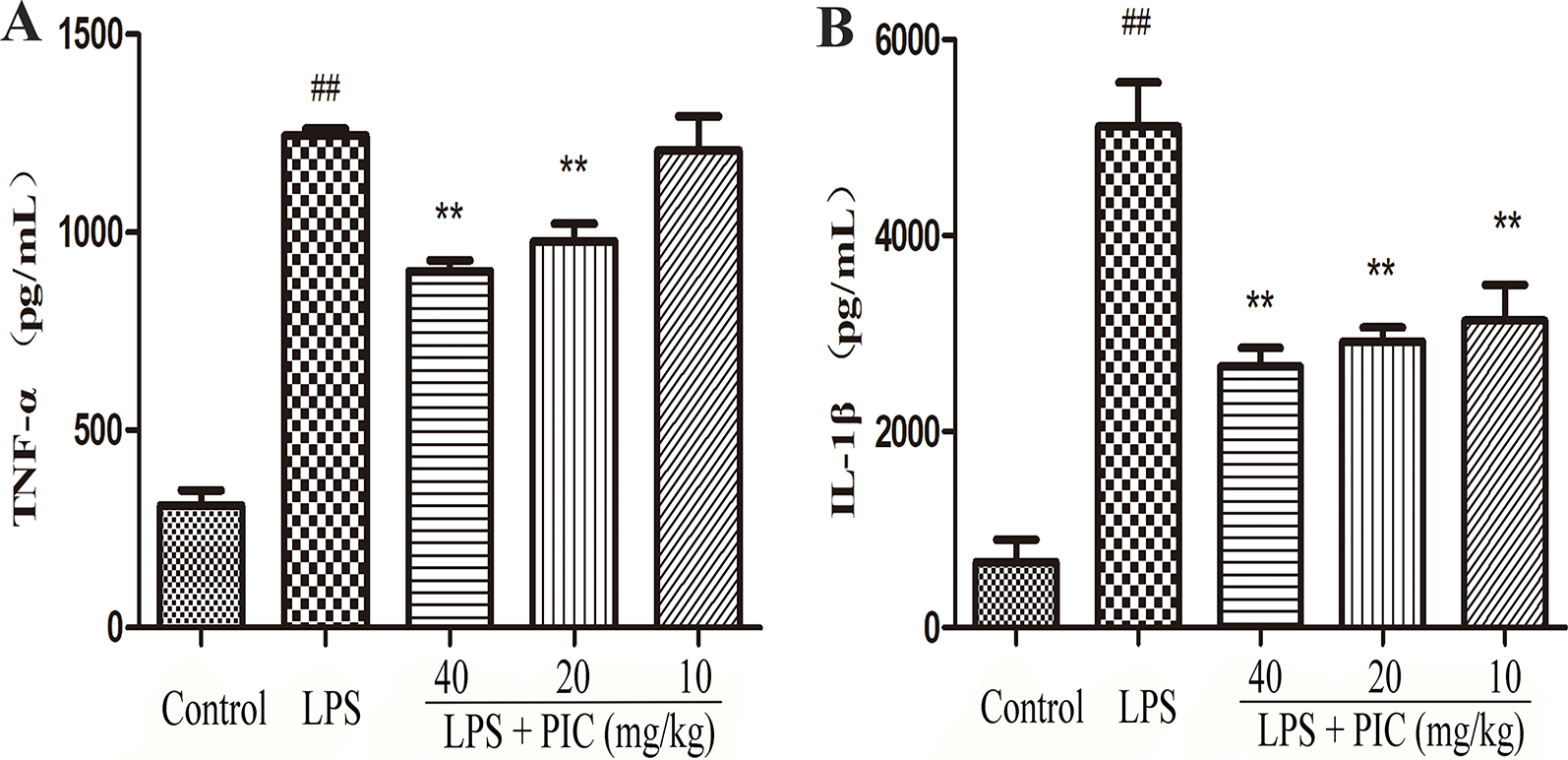
The production of pro-inflammatory cytokines in lung tissues. Lung tissues from different groups were gathered and homogenized with phosphate-buffered saline (PBS). The supernatant was used to analyze the concentration of **(A)** tumor necrosis factor (TNF)-α and **(B)** interleukin (IL)-1β using ELISA kits. ^##^ *p* < 0.01 is significantly different from the control group; **p < 0.01 are significantly different from the lipopolysaccharide (LPS) group.

As **Figure 6** showed, the levels of TNF-α and IL-1β in LPS group were higher than those in the control group. However, the increase of TNF-α and IL-1β in BALF was obviously reduced by PIC in a dose-dependent manner.

### Piceatannol Inhibited the Messenger Ribonucleic Acid Expression of Inducible Nitric Oxide Synthase and Cyclooxygenase-2 and Increased the Expression of Interleukin-10 in Lung Tissues Against Lipopolysaccharide

**Figure 7:**
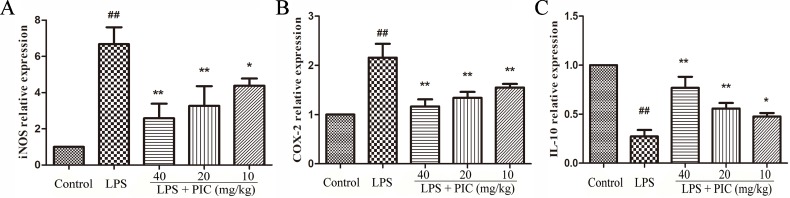
The messenger RNA (mRNA) expression of **(A)** inducible nitric oxide synthase (iNOS), **(B)** cyclooxygenase (COX)-2 and **(C)** interleukin (IL)-10. The transcriptional levels of iNOS, COX-2, and IL-10 were analyzed using quantitative real-time (qRT)-PCR. ^##^ *p* < 0.01 is significantly different from the control group; *p < 0.05 and **p < 0.01 are significantly different from the lipopolysaccharide (LPS) group.

The effect of PIC on the mRNA expression of iNOS, COX-2, and IL-10 was detected in lung tissues from different groups. The results showed that treatment of PIC dose-dependently inhibited the expression of iNOS and COX-2, as well as increased the expression of IL-10 induced by LPS (**Figure 7**).

### Piceatannol Restrained the Messenger Ribonucleic Acid Expression of CD11a, CD62L, ICAM-1, and MCP-1 in Lung Tissues Induced by Lipopolysaccharide

**Figure 8:**
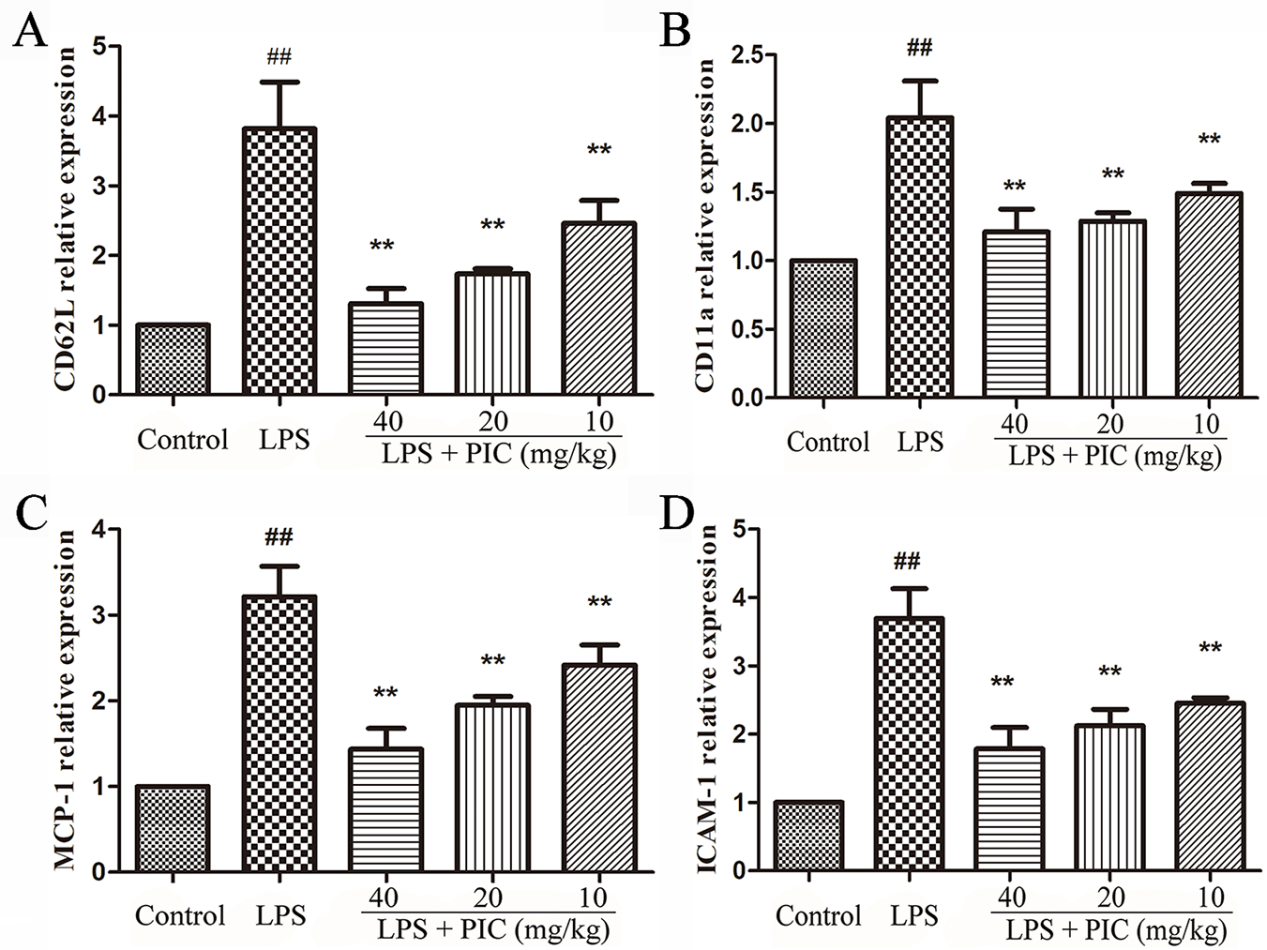
The gene expressions related to cell adhesion molecules and chemokine. The messenger RNA (mRNA) expression including **(A)** CD62L, **(B)** CD11a, **(C)** MCP-1, **(D)** ICAM-1, was examined by quantitative real-time (qRT)-PCR. ^##^ *p* < 0.01 is significantly different from the control group; **p < 0.01 are significantly different from the lipopolysaccharide (LPS) group.

We further measured the gene expression related to cell adhesion molecules and chemokines, including CD11a, CD62L, ICAM-1, MCP-1 in lung tissues. The results showed that PIC significantly inhibited the increase of CD11a, CD62L, ICAM-1, MCP-1 mRNA expression induced by LPS (**Figure 8**).

### Piceatannol Protected the Air-Blood Barrier Against Damage Induced by Lipopolysaccharide

**Figure 9:**
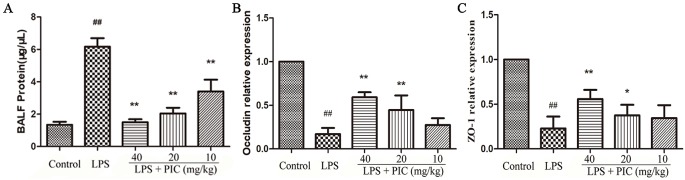
The evaluation of air-blood barrier permeability. **(A)** The total protein of bronchoalveolar lavage fluid (BALF) were measured by the bicinchoninic acid assay (BCA) kits to assess epithelial permeability. The messenger RNA (mRNA) expression of **(B)** occludin and **(C)** ZO-1 was tested using quantitative real-time (qRT)-PCR. ^##^ *p* < 0.01 is significantly different from the control group; *p < 0.05 and **p < 0.01 are significantly different from the lipopolysaccharide (LPS) group.

As the **Figure 9A** showed, LPS challenge increased the levels of total protein in BALF, but the levels of total protein were reduced by PIC in a dose-dependent manner. The tight junction proteins are important symbols for evaluating the air-blood barrier integrity, in which ZO-1 and occludin are regarded as the key marker. In LPS group, ZO-1 and occludin in the lung tissues were significantly down-regulated, while PIC up-regulated the expression of ZO-1 and occludin in a dose-dependent manner (**Figures 9B, C**).

### Piceatannol Inhibited the Activation of TLR4/NF-κB Signaling Pathway

**Figure 10:**
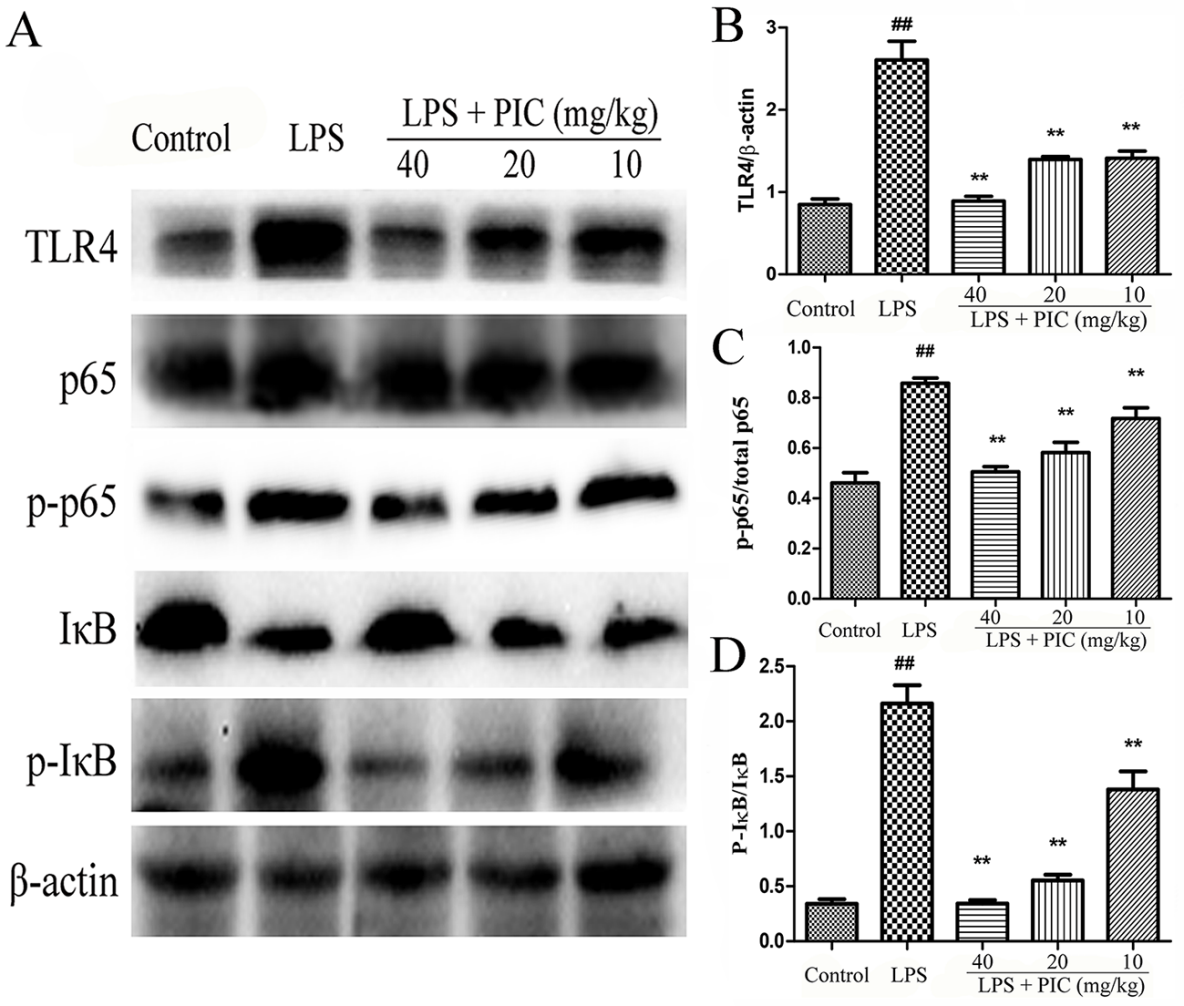
The activation of TLR4/NF-κB pathway in lung tissues. **(A)** TLR4/NF-κB protein samples were analyzed by western blot with TLR4, IκB, p-IκB, p65, p-p65 antibodies. β-actin was used as a control. **(B)** Quantification of TLR4 proteins, **(C)** p-p65 proteins, and **(D)** p-IκB proteins was determined by densitometry. ^##^ *p* < 0.01 is significantly different from the control group; **p < 0.01 are significantly different from the lipopolysaccharide (LPS) group.

With the pro-inflammatory cytokines releasing, the TLR4/NF-κB signaling pathway were consequently activated during the process of LPS stimulation. The results showed that pretreatment with PIC significantly inhibited the expression of TLR4, phosphorylated NF-κB p-65, and phosphorylated-IκBα in the lung tissues induced by LPS (**Figure 10**).

## Discussion

ALI is an acute respiratory syndrome and presents a higher morbidity and mortality (Abraham et al., 2000). However, there is no effective pharmacological approach to cure ALI in clinical practices. Therefore, it is necessary and urgent to develop new agents for ALI treatment. Spleen tyrosine kinase (Syk) is presented in bronchial epithelium (Ulanova et al., 2005). Studies suggested that Syk had ability to regulate the production of IL-6 and ICAM-1 and participated in the development of lung inflammation (Ulanova et al., 2005). Syk was also required for the production of iNOS during inflammatory response induced by TNF, and PIC, a well-known Syk inhibitor, reduced the expression of iNOS induced by TNF (Ulanova et al., 2006). In this study, we found that PIC treatment alleviated inflammatory response by inhibiting the activation of TLR/NF-κB signaling pathway in lung tissues during ALI induced by LPS.

Although inflammation is generally recognized as a defense against various aggressions, excessive of inflammatory response leads to serious tissue damage (Lin et al., 2018). Development of ALI is due to the uncontrolled inflammation which is connected with the over-production of various inflammatory mediators produced by diversified inflammatory cells (Lee et al., 2010; Nie et al., 2019). LPS, a major element of gram-negative bacterial cell walls, is generally recognized as the key factor to induce ALI. Challenge with LPS can result in inflammatory cell infiltration, generate a large number of pro-inflammatory cytokines, and then cause the lung tissues damage (Huang et al., 2018; Ding et al., 2019). In the present study, we found that treatment of PIC significantly reversed the increases of cell infiltration and the levels of pro-inflammatory cytokines including TNF-α and IL-1β both in lung and BALF induced by LPS.

As well known, over-production of inflammatory cytokine, such as IL-1β, drives both COX-2 and iNOS gene expressions in macrophages, causing the release of PGs and NO (Forstermann et al., 1995; Hori et al., 2001). NO is a highly reactive free radical that participates in many physiological and processes (Cho et al., 2004). In addition, COX-2 and its catalysate, PGs, play important roles in the inflammatory process (Chen et al., 2000). Studies showed that the gene expressions of iNOS and COX-2 were markedly increased during ALI induced by LPS (Singh et al., 2019). Thus, the gene expressions of iNOS and COX-2 may serve as a detection index to evaluate the effects of agents on the inflammation. Our studies manifested that PIC significantly inhibited the production of iNOS and COX-2 expressions in lung tissues induced by LPS.

In addition, the effect of PIC on the mRNA expression of several adhesion molecules and chemoattractant was detected in the present study. The adhesion molecules drive the initial attachment of leukocytes to endothelial cells at sites of tissue damage and inflammation (Lee et al., 2000). CD62L is present in all circulating leukocytes (von Andrian et al., 1993). CD11a has been detected in a variety of cell types, including neutrophils, monocytes, and macrophages (Ahmad et al., 2015). Previous studies also suggested that the expression of CD11a was increased during LPS-induced ALI (O'Leary et al., 1997). In addition, MCP-1 is a member of the β chemokine family, and ICAM-1 expression associates with the production of inflammatory cytokines, such as TNF-α and IL-1β (Lee et al., 2000). MCP-1 and ICAM-1 were involved in the development of ALI (Lin et al., 2010). In this study, we found LPS challenge significantly increased the mRNA expression of CD11a, CD62L, ICAM-1, and MCP-1. However, these changes were significantly reversed by PIC.

Emerging evidence suggested that proteinaceous influx induced pulmonary edema, which in turn led to the injury of the air-blood barrier (Kling et al., 2017). Lung wet/dry ratio is an indicator of pulmonary edema. We found that LPS induced a higher lung wet/dry ratio than that of control mice, and treatment with PIC visibly ameliorated the degree of pulmonary edema due to LPS. In addition, the higher levels of total proteins induced by LPS in BALF were also inhibited by PIC treatment. Furthermore, tight junction proteins serve as important components in keeping the permeability of the air-blood barrier, and are down-regulated in the lung during ALI (Herold et al., 2013; Liu et al., 2014). In our study, we also found that the expression of tight junction protein ZO-1 and occludin were significantly reduced in the LPS treatment group mice compared to that in the control groups mice. However, PIC pretreatment reversed these changes induced by LPS.

TLR4 presents in the surface of monocytes/macrophages and it induces an innate immune response in mammals by recognizing LPS. Once LPS is recognized by TLR4, it leads to activate signal transduction and then induces NF-κB activation (Liu et al., 2019a). NF-κB is a ubiquitous transcription factor and closely related to the transcription of the genes responsible for the expression of pro-inflammatory cytokines (Hoffmann et al., 2002). When activated by agonist, NF-κB p65 phosphorylation and proteasomal degradation of the IκB proteins occur resulting in release and phosphorylation of NF-κB dimmers, which are able to translocate from cytoplasm into the nucleus to regulate pro-inflammatory cytokines including TNF-α and IL-1β expression (Hoffmann et al., 2002; Li et al., 2019). An abundance of evidences indicated that inhibition TLR4/NF-κB signaling pathway activation was a target to treat ALI (Chen et al., 2018b; Tan et al., 2018; Liu et al., 2019b). Studies identified that Syk as an upstream mediator of TLR4 tyrosine phosphorylation upon LPS stimulation, and it is a key molecule in the activation of TLR4 signaling (Chaudhary et al., 2007; Ishizuka et al., 2013). In addition, studies also indicated that Syk was associated with the regulation of early phosphorylation of IκBα. Therefore, in the present study, we detected whether PIC, the inhibitor of Syk, has ability to inhibit the activation of TLR4 and NF-κB signaling pathway in lung tissues during LPS-induced ALI. We found that PIC significantly inhibited the activation of TLR4/NF-κB signaling pathway.

## Conclusion

Taken together, these results provided evidences that PIC had potential protective effects against LPS-induced ALI. The mechanism was related with the inhibition of LPS-induced air-blood barrier damage and TLR4/NF-κB signaling pathway activation. Therefore, PIC may be an effective agent for treating ALI.

## Data Availability Statement

The raw data supporting the conclusions of this article will be made available by the authors, without undue reservation, to any qualified researcher.

## Ethics Statement

The animal study was reviewed and approved by the Institutional Animal Care and Use Committee of Jilin University.

## Author Contributions

Conceptualization: B-DF, L-YP, H-QS. Data curation: MY, H-TS, J-HL, J-NH. Supervision: P-FY, H-QS. Writing—original draft: L-YP, B-DF, KS.

## Funding

This work was supported by the National Natural Science Foundation of China (no. 31972724 and no. 31372470) and the Special Fund for Agro-scientific Research in the Public Interest (201403051).

## Conflict of Interest

The authors declare that the research was conducted in the absence of any commercial or financial relationships that could be construed as a potential conflict of interest.
