# Supplementary material for: Protective Effect of Piceatannol Against Acute Lung Injury Through Protecting the Integrity of Air-Blood Barrier and Modulating the TLR4/NF-κB Signaling Pathway Activation
Source: Front Pharmacol. 2020 Jan 22;10:1613. doi: 10.3389/fphar.2019.01613 (PMC6988518; doi:10.3389/fphar.2019.01613)
Supplement: Supplementary file 1 [file Table_1.docx]

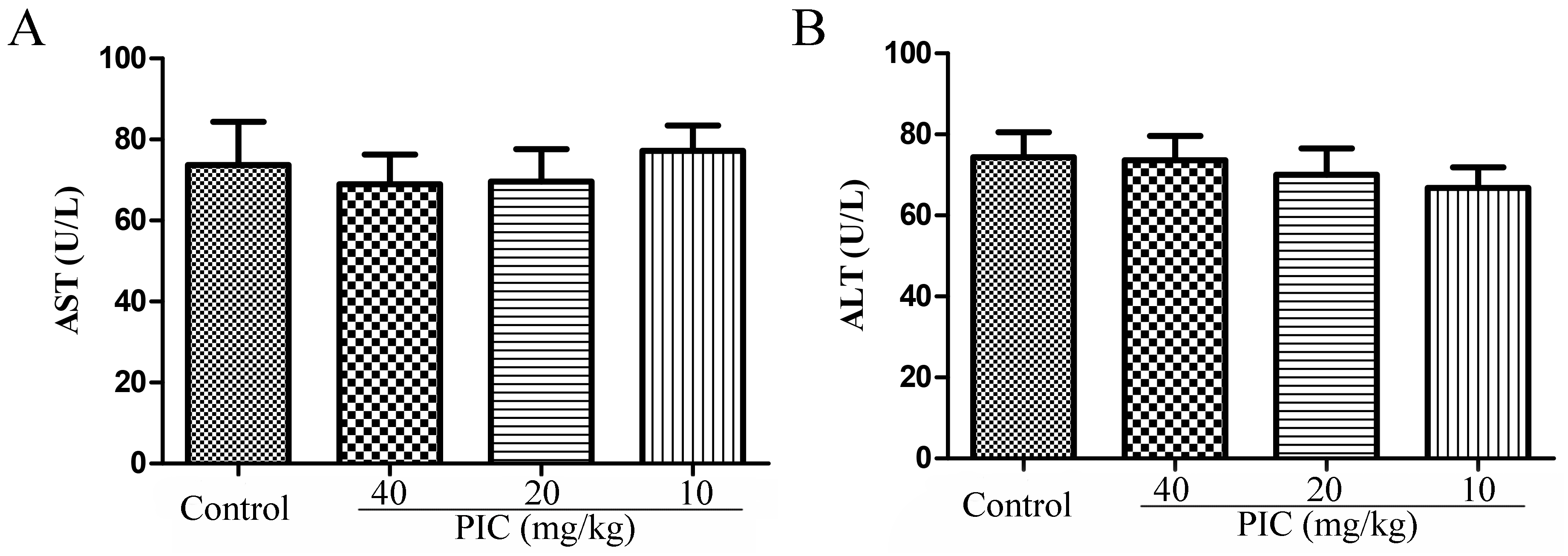
S.Fig.1 The effects of PIC on AST and ALT in serum. After PIC was intraperitoneally injected for 24h, the mice were euthanized and the serum was collected to measure the (A) AST and (B) ALT using commercial kits purchased from Nanjing jiancheng Bioengineering Institute (Nanjing, China).
